# Queer decisions: Racial matching among gay male intended parents

**DOI:** 10.1177/00207152221102837

**Published:** 2022-06-18

**Authors:** Marcin Smietana, France Winddance Twine

**Affiliations:** University of Cambridge, UK; University of California–Santa Barbara, USA

**Keywords:** California, England, kinship, LGBTQ parents, queer studies, race, surrogacy

## Abstract

How does race and location shape the reproductive decisions of gay men who are intended parents? In this article, we propose the concept of *strategic racialization* to characterize the ways in which gay male parents employ racial matching in their selection of egg donors and surrogates in the United States and United Kingdom. We argue that racial matching is a strategy of stigma management. This study draws upon interview data from 40 gay male couples who formed families through surrogacy. We find that pre-conception fathers seek racialized resemblance to reinforce kinship between themselves and their children. In California and England, gay men seeking donor eggs engage in racial matching, which reveals that the racialized biogenetic model of kinship remains dominant. This study makes a significant contribution to the literature on race and queer family formation.

Gay intended parents employ racial logics as they evaluate the eggs and sperm available on the fertility market. In other words, gay intended parents—like other families—negotiate the meaning of “race” when evaluating what constitutes a “good” or “good enough” match for them. In this article, we draw on transnational research in California and England,^1^ to provide an analysis of the meanings of “likeness” and the racial logics employed by single men and gay couples who are forming families using surrogacy and egg donation. We understand racial logics as specific uses of racialization that individuals link to broader racialized social structures. Although whiteness is a privileged socio-racial status in both California and England, its boundaries and definitions differ in California^2^ when compared to England. In the local contexts of racism and stigma due to transgression of racial, sexual, and reproductive norms, we ask how gay men assert their constrained agency as they negotiate their imagined future children through surrogacy arrangements in these two locations.

In this article, we introduce the concept of “strategic racialization.” We then review the literature on the use of “racial matching” by lesbian and gay intended parents in their choice of gamete donors. After detailing the research methods, participants, and the research sites, we provide an analysis of how gay male intended parents made “queer decisions” in diverse local, and national contexts.

## Strategic racialization

In this article, we build upon concepts and an analytical lens introduced by France Winddance Twine (2010), a North American sociologist in her work on British interracial families. In *A White Side of Black Britain: Interracial Intimacy and Racial Literacy*, an ethnography of transracial families in Leicester,^3^ Twine employed ethnographic case studies to analyze the ways that White mothers negotiated the stigma that was transferred to their mixed-race children born in interracial relationships. Twine argued that the meaning of whiteness, blackness, and “mixedness” was negotiated in the context of working-class whiteness being a stigma for Black families’ members, and blackness and “mixedness” being a stigma for White families’ members. Thus, what constituted transgressive behavior differed between the aspiring Black family members and the White family members of working-class origin. One of Twine’s central arguments was that “whiteness was a contingent form of capital” which was determined by the marital status, class background, and cultural tastes of the White mothers raising children of African-Caribbean heritage. Developing a concept of racial literacy (Twine and Steinbugler, 2006), Twine (2010) showed how White parents negotiated stigma that was associated with their perceived “character” and the transgressions of racial norms they made as parents of children born in interracial heterosexual relationships. Following Twine (2010), we understand a transgression as non-normative behavior whereby (intended) parents challenge dominant kinship norms, such as racial purity (e.g. through establishing interracial families), or, in our study, also heteronormativity (as families with gay fathers) and attribution of motherhood to the person who gives birth (e.g. through surrogacy) (pp. 60).

Our theoretical approach and the analytical case study methodology are also inspired by Charis Thompson’s (2005) *Making Parents: The Ontological Choreography of Reproductive Technologies.* Thompson showed how fertility patients in the United States naturalized their choices of gamete donors and surrogates. In the analysis of what she called *strategic naturalization*, she used family case studies in order to show how each one of them mobilized meanings of biology in different ways in order to “disambiguate kinship.” Whether they naturalized their family arrangements by emphasizing their genetic, gestational, or other links to children, they invoked biological categories which they imbued with cultural meanings. The fertility patients that Thompson interviewed looked for “genetic similarity” in particular with gamete donors. For the intended parents, “genes were coding for ethnicity” and for specific racial distinctions which they sought to share with the donors (Thompson, 2005: 157, 168). One of the main reasons for which the women and men in Thompson’s study employed this kind of *strategic naturalization* was to manage stigmatization they could face due to revealing their infertility (Thompson, 2005: 118).

According to this logic, intended parents who use reproductive technologies feel that the transgressions they make expose them to heightened social scrutiny. This logic was also characterized by Sarah Franklin and Celia Roberts (2006) in their study of preimplantation genetic diagnosis (PGD). The heterosexual intended parents they interviewed felt they had to account to the wider society for their decision to use PGD. This reproductive *accounting for PGD* meant that “prospective PGD couples are asked to give a robust account of their desire to pursue PGD.” However, accounting for the use of a novel technology is only one part of the heightened “reproductive accounting” that LGBTQ + parents often need to undertake. They may need to account for their decision to pursue parenthood not only due to technological transgression, but also in the face of homophobia and racism.

The interviewees in our study strategically mobilized racialized categories in their pre-conception decisions. Building on Twine’s, Thompson’s, and Franklin’s work, we introduce the term “strategic racialization” to illuminate the ways in which gay men engage with race to manage stigma that may result from transgressions they make or not during racial matching in egg donation, alongside the transgressions as gay parents and parents through surrogacy. We define “strategic racialization” as the use of racialized categories by intended parents in order to make their families look more “natural” and “normal” in the context where they live, and through this to account for the transgressions they make as parents through surrogacy and gay parents.

In the process of strategic racialization, the pre-conception parents interviewed in this study used racial matching in the choice of gamete donors first of all as a “kinship device.” This is in agreement with previous literature, which we review below. Second, the gay men in our study also engaged in racial matching as a strategy of pre-conception stigma management, as was suggested by literature on lesbian mothers. We provide new comparative data on racialization in families of gay male parents from the United Kingdom and United States, and we ask about their reasons for racial matching and potential differences or similarities between both locations.

Our data show that both in California and in England, gay male parents of different racial identifications use racial matching as a proxy for kinship. Thus, the men in our study (White, Black, Brown Latino, Asian) *usually* chose the egg donor whose skin color resembled their own. This is in line with the traditional Euro-American kinship norms, whereby racialized resemblance is thought to be inherited through genes, and it codes for kinship (Franklin, 1997: 21, 51; Thompson, 2005: 157, 168). On the contrary, we show that in some cases, racial matching can also be mobilized by gay men as a way to negotiate and counter racism and homophobia. In those cases, location-specific motives may come into play, such as belonging to the racial majority or minority where they live. Such location-specific motives, however, seem to be subordinated to the overarching use of racialization as a kinship device. This suggests the continued relevance of the Euro-American kinship norm of racialized biogenetic kinship. It occurs despite local differences, and despite kinship transformations created by gay men using surrogacy.

## Racial matching as a kinship device

We contribute a much-needed case study that provides transatlantic data on gay male family formation and makes a significant empirical and theoretical contribution to queer studies, sociology of reproduction, and sociology of race—specifically whiteness studies. Earlier research on the use of assisted reproduction and surrogacy by men in the United States and United Kingdom has not included a comparative analysis of the ways that gay men employ racial ideologies to strengthen kinship ties (Murphy, 2015; Norton, 2018). Earlier studies did center racialization in the analysis of kinship strategies by gay men. Dean Murphy found that many of the participants in his study (including interracial/multiethnic couples) matched the non-genetic father’s perceived race to that of the egg donor. Murphy (2015) interpreted it as “playing strategically with the phenotype in order to create kinship through what they understood to be a visually coherent family unit” (pp. 217).

On the other side of the Atlantic, Wendy Norton (2018) found that gay male intended parents sought egg donors that would resemble them “to create a semblance of a genetically-related two-parent family” (pp. 244, 156). Some of them also hinted at resemblance in terms of their race (e.g. “Caucasian”) but they did not problematize it. This could be explained by the fact all participants but one in Norton’s study were White, so they may have treated race, as well as its transmission through genes, as obvious and “unmarked” (Frankenberg, 1993). Similarly, White lesbian women in Ryan and Moras’ (2017) US study also placed emphasis on matching certain physical/ethnic characteristics, without specifically mentioning race.

In this article, we build upon earlier research to advance the empirical and theoretical debates in the field. The use of *strategic racialization* by gay male intended parents allows us to theorize how in these family forms there is consistency in perceptions of race as biological rather than a socio-political category. That is, gay men who employ egg donation and surrogacy on both sides of the Atlantic conceptualize race as a form of property, a privileged asset, a genetic possession that remains a crucial component of kinship. This fits in with the traditional Euro-American kinship model, where biogenetic ties between heterosexual parents and their children were thought to give rise to kinship, that is, to “diffuse, enduring solidarity” (Franklin, 1997: 21, 51; Schneider, 1968). Our findings also echo earlier research by Black, Indigenous, and critical race scholars, who have pointed out that the settler model of Euro-American kinship in America involves the biogenetic understanding of race, whereby genes code for race (Thompson, 2005: 157, 168). Within the traditional Euro-American kinship model, standard families were racially homogeneous, and the reproduction of white privilege was policed by normative ideas of “racial purity” (Roberts, 1997; Russell, 2018; TallBear, 2018; Twine and Smietana, 2021). Scholars have argued that gay men’s families remain embedded in compulsory heterosexuality and colonial legacies that reproduce racialized versions of Euro-American kinship. Gay parents have been found to employ different strategies that deflect from the impact that racialization could have on their families’ lives (Keaney, 2019; Nebeling Petersen, 2018).

Our findings on the use of race by gay fathers through surrogacy in the United Kingdom and United States overlap with findings from other locations, including on lesbian mothers and heterosexual parents. In different locations such as the United States, United Kingdom, Australia, and Scandinavia, scholars have noted that intended parents, including lesbian and gay parents, often seek likeness between themselves and gamete donors (Mamo, 2007; Murphy, 2015; Newman, 2019; Nordqvist, 2012; Ryan and Moras, 2017; Thompson, 2005; Weston, 1991; Twine and Smietana, 2021). And likeness is racialized and conceived in terms of eye color, hair color, hair texture, skin tone, and facial features. Weston (1991), Mamo (2007), Ryan and Moras (2017), as well as Newman (2019) in the United States, and Nordqvist (2012) in the United Kingdom have found that lesbian women chose sperm donors who resembled them in racial terms, in order to signify kinship between the mothers and the future child. The interracial couples usually matched the sperm donor’s perceived race to that of the non-genetic mother (Mamo, 2007), or to the sibling’s race (Newman, 2019). Mamo (2007: 206) characterized this process as “a kinship device” that “enables the recipients to envision their own social connections to the imagined children.” In the United States, United Kingdom, and Sweden, intended parents have also been found to use racial matching in contexts where it is encouraged or enforced institutionally by sperm banks (Quiroga Szkupinski, 2007), egg banks (Davda, 2018), and fertility clinics (Dahl, 2018). However, implicit in the parents’ decisions may have also been the question of their perceptions of potential stigmatization, and this question was often not the main focus of previous literature.

## Racial matching as pre-conception stigma management

In *Stigma: Notes on the Management of Spoilt Identity*, Erving Goffman (1986 [1963]) provided an analysis of how people negotiated different types of stigma. In his analysis, stigma was relational, and homosexuality was one type of stigma. Although our study has been inspired in part by Goffman, in the 21st century, and in some locations where same-sex marriage is now legal, gay male parents are usually not negotiating that same type of stigma. Among different kinds of stigma, Goffman (1986 [1963], edition: 4) also characterized the “stigma of race, nation, and religions, these being stigma that can be transmitted through lineages and equally contaminate all members of the family.” Below, we refer to the literature on queer family formation that shows that both kinds of stigma—sexuality and race—are still relevant to intended parents’ pre-conception decisions, as well as a new kind of stigma: use of surrogacy.

In *The Presentation of Self in Everyday Life*, Erving Goffman (1959 [1956]) presented his theory of dramaturgy in which individuals perform a role, as if on stage with props. In this case, children could be thought of as a type of kinship prop. For intended parents, the appearance of children is evaluated and could result in one being discredited as biological kin. Gay men, like all parents, must negotiate their ability to protect their children (and themselves) from being “discredited” as natural—that is biologically related family members. One mechanism employed by gay men is to try to establish physical resemblance with their children. In other words, they seek egg donors whom they hope will produce a child who will share those features valued by their natal family and prized in the local and national context. We bring Goffman’s concepts into dialogue with the contemporary literature on queer studies and whiteness studies. Specifically, we ask if in the context of English and Euro-American kinship, a lack of racial homogeneity within a family is perceived as a potential source of stigmatization or marginalization.

How do gay male couples manage the meanings of race and stigma as pre-conception parents? Gay and lesbian parents identify the stigma their children and themselves might face if they were perceived as too transgressive. In other words, they are aware of the stigma they could face if they transgressed the racial boundaries. Therefore, they undertake what could be called pre-conception stigma management. The issue of stigma has been undertheorized in the literature on queer family formation. In this article, we build upon Smietana’s (2016) earlier research, in which he interviewed intended gay male parents in Spain. As they were forming families with the help of egg donors and gestational surrogates, they employed racial matching. They said that they consciously used racial matching in order to shield their children from potential stigma, as well as to emphasize the “naturalness” of the family. Also American and European gay men, who were interviewed by Smietana (2017, 2019) during their surrogacy arrangements in the United States, expressed a fear that their families could be marginalized due to homophobia. Twine (2006, 2010) found in her research on British interracial couples that the White parent was challenged publicly about the “naturalness” of her family and the mixed-race children were stigmatized. Smietana and Twine, working in different national contexts with couples perceived as transgressive by members of the dominant group in terms of sexuality and race, both found that what Goffman (1986 [1963]) called “impression management” was a central part of parenting.

Gay and lesbian parents in some national contexts are aware that they may face stigmatization as the parents of young children. In her research on Australian gay male couples in Thailand, Andrea Whittaker (2019) found that gay men had to negotiate stigma that is attached to non-traditional family units. Whittaker (2019) writes, “[t]he accusations leveled at Peter, of exploiting women and denying children a mother, point again to gendered politics gay couples face undertaking surrogacy . . . They point to the deep emotions perceived threats to heteronormative norms evoke for some people” (pp. 81).

In addition to transgressive family forms involving gay male parents, racial transgressions are also central to the pre-conception calculations undertaken by gay parents. In a study of lesbian women who sought to have children in the United Kingdom, Petra Nordqvist (2014) found that “[m]any white couples . . . indicated that they avoided selecting a donor who was perceived to be of a different ethnicity because the child might then encounter racism as well as homophobia” (pp. 651). In a demographic context that consists of the White majority, in particular “[t]he white couples approached donor selection as a practice that could help them ‘fit in’” (Nordqvist, 2012: 651). Similarly, in her study of lesbian mothers in the United States, Laura Mamo (2007) found that some of the women she interviewed understood racial matching to be both a kinship device and a strategy to counter stigmatization (pp. 191, 207).

## Donor catalogs and racial hierarchies

Intended parents use racial matching in the context of post-colonial racial hierarchies. In the United Kingdom, as well as in the United States and other former British colonies, whiteness is a form of cultural, social, and symbolic capital, which provides many lifelong advantages (Ahmed, 2006; Frankenberg, 1993; Nordqvist, 2012; Twine, 2010). In *White Women, Race Matters: The Social Construction of Whiteness*, Ruth Frankenberg (1993) conceptualized whiteness as a set of linked dimensions: “a location of structural advantage, of race privilege”; a standpoint from which white people look; and “a set of cultural practices that are usually unmarked and unnamed” (pp. 1).

The reproduction of whiteness is sustained by ideologies of racial purity (Andreassen, 2017). For this reason, fertility clinics and recipients in the United Kingdom (Davda, 2018), the United States (Quiroga Szkupinski, 2009), and Scandinavia (Dahl, 2018; Homanen, 2018) have been found to police the boundaries of whiteness and discourage interracial gamete donation, in particular for White recipients. For example, Priya Davda (2018) found that in the United Kingdom, “recipients were willing to accept a donor with lighter skin tone than themselves but not darker skin tone” (pp. 310). Similarly in Finland, Riikka Homanen (2018) demonstrated that “the logics of matching protect the ‘purity’ of whiteness but not of brownness or blackness” (pp. 28). Also at the transnational level, Amrita Pande (2021) observed very similar racial logics in the ways that eggs provided by White donors from South Africa are acquired in international clinics elsewhere (pp. 337). She found that “the transnational fertility industry naturalizes choices that reaffirm the desirability of whiteness.”

These racial logics are exacerbated by the ways that race becomes commodified by the fertility industry, in the process of “racial commodification” (Deomampo, 2019). A prime example of this is the existence of drop-down menus for race and ethnicity on egg and sperm bank web pages. According to Camisha Russell (2018), they are “a stark reminder of the deep social practices that have divided and continue to organize people in terms of race” (pp. 160). Yet on the contrary, Russell (2018) argues we need to respect the fact that “no matter what their history, the categories of race and ethnicity can have rich cultural meanings” for intended parents who opt for donors of a given race (pp. 161).

As eggs are racialized and commodified, their providers and recipients conflate desires for similar appearance with beliefs about a “cultural background” they share with donors, the “racial purity” of donors, and the eggs’ ability to carry inherent racial qualities (Deomampo, 2019; Thompson, 2005). As Daisy Deomampo (2019) showed in her study of Asian egg donation agencies in the United States, “marketing and matching practices in egg donation perpetuate stratified norms of race and class, creating hierarchies of value based on notions of purity and market demand” (pp. 630). These hierarchies of value are only exacerbated by the fact that “the legal and normative context turns eggs into capital,” as Lisa Ikemoto (2009) argued.

The global reproductive industry sustains such hierarchies of value. An example of this is commercial gestational surrogacy with egg donation in the United States, which was used by all the 16 families interviewed in the United States in our study. Also in the altruistic surrogacy model that is legal in the United Kingdom, most intended parents resort to commercial egg donation. Among the 24 families interviewed in the United Kingdom, five pursued altruistic egg donation (their surrogates used their own ova), and 19 pursued commercial egg donation.

In this article, we show how gay male parents understand and mobilize racial hierarchies as they engage with the reproductive industry of egg donation. Race is a modern idea that was created during European colonialism to restrict, deny, or provide access to rights such as land ownership, voting, citizenship (Almaguer, 1994; Fredrickson, 1981, 2002; Smedley, 1993). A number of groups today considered White in the United States were not White in the 19th century, including Italians, Irish, Greeks, and Jews. European immigrants who were not “White on arrival” re-positioned themselves and achieved whiteness (Jacobson, 1999). The varieties of whiteness were produced by differences in religion, language, and skin tone (Thompson, 2009). The socio-political category of White continues to be expanded and modified (Twine et al., 1992; Root, 1992, 1996; Streeter, 2012; Warren and Twine, 1997). In the case of Hispanic/Latino, race is an “elastic” category that can include people who self-identify as Black, White, or Multiracial. In the United States, on the Census, the category Hispanic is not a “racial category” and it reflects the fact that this term accommodates people who self-identify along the racial spectrum, including those who identify as White and are socially perceived as White as well as those who identify as Black or Brown. In a recent census, more than 50 percent of people who identified as Hispanic, identified their race as White. Therefore, in this article, we ask what the intended parents mean by different racial categories when they mobilize them in their family making projects, as well as how those categories might be understood by others in a given context.

## Research methods and participants

Between 2015 and 2021, one of the authors (Smietana) conducted 40 interviews with gay singles and gay couples in California and England. The anonymized interviews were analyzed and the article was co-written together with the other author (Twine). Twine, based in California, provided expertise in critical race theory, and Smietana, based in England, offered expertise in queer studies. This collaboration was part of a larger project (Smietana et al., 2018; Twine and Smietana, 2021).

In both research sites, several methods were employed to recruit participants, including (1) online posts and notices on community forums, (2) attendance at events at organizations including *Men Having Babies* and *Our Family Coalition* (in the United States), and *Surrogacy UK* and *Two Dads UK* (in the United Kingdom), (3) contacting local fertility clinics and surrogacy agencies, (4) participant observation and accompanying interviewees to clinics and surrogacy-related events such as agency anniversaries, and (5) snowball sampling. Among the 40 families who participated in this study, 16 were interviewed in California and 24 in England. Interviews in California were conducted in 2015 and 2016 at several sites including the participants’ homes or in public cafés, and follow-ups and further interviews were conducted in 2021 via video-calls. During the 2020–2021 pandemic, interviews in England were conducted online via Zoom and Skype. All interviews were semi-structured. Email communication was used to schedule follow-up interviews with selected couples. In the analysis for this article, the focus of comparison was the men’s understandings of race and ethnicity, and how they were situated in their stories of family creation.

The participants in the study were men who identified as gay. They included pre-conception parents and parents of children conceived through surrogacy. They were between the ages of 24 and 52 years old. Among the 40 families in this study, 32 self-identified as White and eight were interracial couples. In each interracial family, one partner self-identified as White, and the other self-identified as non-White and, respectively, Japanese American, Latino, Filipino, Brazilian, British Caribbean, Black British, and South Asian in two cases. Among the 40 families, 36 were couples (either married or in civil partnerships), and four were single parents. All of the participants were university educated and most had also earned post-graduate degrees. Most were professionals. Thus, they were middle class and had the economic and other resources to enter into surrogacy arrangements.

## Research sites

Field research including on-line and in-person interviews was conducted in two sites: England and California. In England, where altruistic surrogacy is a legally recognized pathway to parenthood, interviews were conducted in London and southern England. The United Kingdom is one of the few jurisdictions in Europe where altruistic surrogacy is legal and has been available since 1985, to gay couples since 2010, and to single parents since 2019.^4^ Some regions of the United Kingdom, such as London, Manchester, and Leicester are multiethnic and multicultural. Post-World War II, immigration from former colonies dramatically increased the ethnic diversity and changed the demographics in Britain’s major port cities. However, people who identify as White remain idealized and comprise the racial and political majority.^5^ For example, in England and Wales, where the interviewees came from, 86 percent of the population is White.

California, a state with 40 million residents, has the largest population in the United States, followed by Texas. California also has the largest population of people classified on the US census as Hispanic or Latino of Mexican origin. In addition, in many parts of the state Mexicans comprise an ethnic majority, and thus Whites are predicted by many to be an increasingly smaller percentage of the state population. Only 36.5 percent of California residents self-identify as “White alone, not Hispanic or Latino,” and 39 percent identify as “Hispanic or Latino.”^6^ California is also one of the birthplaces of the Gay Pride Movement. With highly visible and politically powerful multiethnic LGBTQ communities, the San Francisco Bay Area is an ideal site to examine queer family formation. Since the late 20th century, California has been a global destination for commercial surrogacy and services targeting the unique legal needs of the LGBTQ community.

The average cost of a commercial surrogacy arrangement in the United States was $130,000. All the arrangements in the US sample were gestational (i.e. two women were involved: a surrogate and an egg donor). In the United Kingdom, only altruistic surrogacy is legal, yet surrogates are compensated for the necessary expenses, and additionally, commercial fertility clinics are used in the egg donation process. In the UK sample, among the 24 families, five used traditional surrogacy where the surrogate used her own ova so no other woman was involved as an egg donor. Therefore, the cost of a surrogacy arrangement in the United Kingdom ranged from £12,000 in case of traditional surrogacy up to almost £60,000 in case of gestational surrogacy with egg donation in a fertility clinic. Both in the United Kingdom and in the United States, the men drew on their own salaries and savings, loans including mortgage, as well as support or inheritance from their own parents. Some of the men were also involved in some forms of LGBTQ activism within transnational and local surrogacy and gay family associations.

In the next sections, we will introduce four couples that represent the variation in family composition (mono-racial or multiethnic) and the racial logics found in the larger study. We will also include comparisons to other study participants. Through our analysis of their “queer decisions,” we aim to illuminate the complex pre-conception calculations that gay men make as they manage presumed stigma and reconcile their desire for “resemblance” or likeness.

Couple 1, Mike and Bob, are a White couple living in California. They represent the majority of the study participants, as 32 out of the 40 interviewed families were identified as White and followed a similar racial logic, whether they lived in California or England. It was the logic of racialized resemblance between the father(s) and the child(ren).

Couple 2, Jason and Max, are a multiethnic couple (Japanese American and White American) living in California. And Couple 3, Luke and Jack, are a multiethnic couple (brown-skinned Filipino and White Australian) living in England. Both of these couples have multiethnic and interracial heritage, and both of them follow a similar racial logic—which they also share with five out of six (all but one) remaining interracial couples in the study. Indeed, it is also the logic of racialized resemblance between the father(s) and the child(ren), just like in case of the White Couple 1.

Couple 4, Kevin and Arthur, are a multiethnic couple (brown-skinned Latino and White British) living in England. They follow a unique racial logic of resemblance between the child(ren) and the racial majority group in a given location. Some of the other study participants also mention this racial logic, for example, Couple 2 in California. This logic may reveal important local differences, given the racial majority in California is different than in England. However, in no other case is this logic as clearly pronounced and decisive as for Couple 4, Kevin and Arthur in England.

## White like me: a monoracial couple in California

Mike,^7^ a 44-year-old head of Human Resources and his husband, Bob, a 40-year-old surgeon, are a White professional couple. They met in Los Angeles and subsequently moved to the San Francisco Bay Area, as a couple. They have been married for 8 years and are the parents of two boys between the ages of 2 and 5. Their third child is currently being carried for them by a surrogate. They identify as Catholics and attend their local church every week—which they described as “a very Bay Area Catholic” church, where they were fully accepted. For example, their priest invited them to baptize their children there. Mike and Bob have also been involved in activism in support of LGBTQ family inclusion, by participating in panels organized by *Men Having Babies*, or talking about LGBTQ family rights in work settings.

Mike, a native of Wisconsin, identifies as of German and English heritage. Bob, a native of California, identifies as White and is of Colombian and Irish heritage. They used the same egg donor, and they alternated as the genetic fathers. They chose which embryos were viable. As a result, Bob became the genetic father of their first child, and Mike was the genetic father of their second. Their main criterion was choosing an egg donor who would physically resemble them. A secondary criterion was not genetic but it was the social and educational status of the donor. They thought physical resemblance would make it easier for their own children and for other people around them to recognize them as a family:

Bob: So, for me it was about educational level and intelligence, and I think Mike was more interested in just overall looks.

Mike: *I wanted us to have a family that looked like us*. We were looking for linkages, so I’m German and English. You’re Colombian and Irish . . .

Bob: And so, we tried to think about what skin tones would we want. We wanted to look like a family. I think we’d really want to have kids ourselves. Obviously, we can’t. So, we did our best approximation of guessing into a crystal ball. (. . .)

Bob: Yes, to look like a family, I guess. Because we know they’re going to have enough issues as it is.

Mike: *Having two dads I think is going to be hard enough*. And *I think it’s just easier to relate*. I don’t know, I just thought forward about teenage kids when they’re really in a difficult time. I don’t know, I just want *when the external world looks at us that it feels like a family. And then, I want our kids to see resemblance in how we are as a couple*, and not be one or the other. (. . .) I wanted it to be our family, and for some reason *resemblance was a way that I thought would be really important to signal to others, and to feel like it was ours*.

Even in an LGBT-friendly context such as northern California, gay men may have negative experiences with their own relatives when they choose to form families. Mike and Bob’s natal families expressed shock by their decision to have children. The couple recalled that at their wedding anniversary, their first surrogate met Mike’s mother and Bob’s mother. The women had an hour-long conversation, which dispelled the doubts that their mothers had about commercial surrogacy. As a result, Mike and Bob were very conscious of the potential stigma that gay male parents could face from their natal families. This explains why they placed emphasis upon physical resemblance “so as to make it easier for their children and themselves.”

Mike and Bob represent the majority of White-identified gay couples in this study. Like all of the couples in the study, they sought physical resemblance or likeness between themselves and their future children. This likeness included features that they racialized, such as the skin tone, eye color, and hair color.

On both sides of the Atlantic, without exception, the White gay men used similar racial logics. We use the term racial logics to refer to their beliefs about the meaning of racial difference and, in case of these men, the privileging of features that are socially identified with European heritage and whiteness. They sought to create the impression that they were biologically related to their future children. In this view, they argued that this would help them minimize questions about their child’s lineage. For example, a White English couple Ed, a 48-year-old software programmer, and his partner, Theo, who is a 38-year-old chef, lived in London and had two children between the ages of 5 and 8 years old. They formed their family with the services of an American gestational surrogate. Ed was the genetic father of one child, and Theo of the other. As Theo recalled,

Our kids already are going to be different. And then, you know, so then it’s going to be “Why have you got two dads?” And then if they look . . . if they are different skin colors, they’ll be like, “Well, why do you have different color skin to your parents?” And they’ll be like, “Oh, we chose it for aesthetics”? Like it doesn’t make any sense, you know? *We want them to look like us. We want the least amount of questions that ask us to validate our own family’s existence.* So we don’t need to make things more difficult for ourselves.

In the next section, we will meet an interracial couple who sought the “California look” for their baby, alongside likeness between themselves and the baby. California, a state with the largest Latino population in the United States, provides a context in which there is a perception that what some call “the Latino look” is ideal and representative. This is complicated by the fact that the US census category defined as “Hispanic or Latino” refers to Spanish surname and can include people who identify across a wide racial/ethnic spectrum. Roughly half of the Hispanics in the United States identify as “White.”

## “The California look”: multiraciality as the ideal look

Jason, a 35-year-old White, and Max, a 36-year-old Japanese American are a middle-class professional couple, who met in 2000, and got married in 2012. They live in the San Francisco Bay Area, a region in the United States which is celebrated for being a welcoming and utopic space for LGBTQ families. Jason was born in Florida and grew up in rural North Carolina, and Max comes from southern California. Max is employed as a medical doctor in the local public hospital, and Jason works as an administrator in a medical company.

Like other gay male couples in this study, Jason and Max had long thought about forming a family with their own children. Until recently, they did not have the economic resources to pursue what is an expensive pathway to parenthood. As they said, “We were much younger, and did not have any means to do anything of course. And so it was always very sort of abstract, in the future.” Thus, this method of forming a family is not affordable to all gay male couples. Even with their professional jobs, Jason and Max needed to take a mortgage loan in order to pay for their surrogacy expenses.

Following a medically unsuccessful attempt with Jason’s sister, also White American, as an egg donor with Max’s sperm (genetic father), they chose a “mixed-race” egg donor on the commercial anonymous donation market recommended by their clinic. They decided that they wanted a child that was perceived as mixed-race: “Asian mix” or “White Asian,” in their own words. The men wanted their future child to reflect the multiracial lineage that they represented as a couple. From their perspective, it would be “easier” for the child. This is an argument that was also employed by White and Black family members opposed to interracial family formation. As Jason said,

We were kind of looking for someone who might be a mix, like an Asian mix, maybe White-Asian. Because of course Max and I are different races. He has Japanese descent, I’m a White guy and with the prospect of each of us contributing sperm, there was the possibility that our children could look really nothing alike, even if they had the same egg-donor . . . We thought if we had an egg donor who was mixed-race, that the children would both be mixed and that might make things a little easier.

This way, the couple said, it would be easier for the child to feel they belong to the family, and it would be harder for other people to question their mutual family ties. Since they were also planning on having a second child in the future, they had decided to each be the genetic father of one of the children. Their logic was based in part on the age difference between the two of them. In pursuit of their goal of having two children who were genetically related to both parents, they had agreed to use the same egg donor of multiracial heritage so that their children would also physically resemble each other. Here we see the logics of likeness. In addition to likeness between the parent and child, in this case two fathers socially classified as racially distinct, we also see another form of likeness being sought: between the siblings.

The men described how they managed their anxieties about likeness and why they mattered. They quoted a recent media story about a child that had been taken away from gay men who were approaching the end of the adoption process. Jason and Max argued that a genetic link between one of the men and the child would have resulted in a successful adoption. In addition, likeness between the child and the fathers could protect their families—that is, decrease their vulnerability in public. Jason and Max’s emphasis upon a genetic connection was motivated not only by a fear of their marginalization as intended parents, but also a fear of the child’s marginalization or even bullying due to having gay dads. They believed that a shared physical resemblance would help the child feel they belonged to the same family. To illustrate this point, Max told several anecdotes about growing up with half-brothers from his father’s previous marriage. Having siblings who were “very different in lots of ways” contributed to Max’s perception that having a different skin color among family members was problematic:

I also knew someone who had adopted a child from China. He was Caucasian, he was White, and he just brought the baby home from China. And soon after that, he told me the story about one of the kids coming up to him and saying “how come your baby doesn’t look like you?” And he thought, the first thing in his mind was “oh God, it’s starting already, I thought I would have a few years before I had to deal with this.” I mean you can’t control everything that happens to your child by no means. But some things you kind of understand as being important. I guess that does play into it, *thinking about the child having to navigate yet another difficulty. One is having two gay dads*.

Their desire to minimize their children’s and families’ discrimination inspired Jason and Max to seek an egg donor of multiracial heritage. While race is a social category and many children produced by “biological” parents in heterosexual families do not look alike, racism and homophobia have real consequences.

Jason and Max also sought likeness between the child and the society they were living in. It was the diverse Californian society, consisting of a myriad of racial and ethnic backgrounds. This way Jason and Max, as a mixed-race couple, also reflected the dominant mixed-race composition of California. They chose a mixed-race egg donor, who was part Filipina and part Swedish:

From the photo we have of her she just looks like a Californian to me (. . .) There’s this California look that for me has emerged, that reminds me a bit of Brazil. You could come from all over the world to live here and the next generation looks like a mix.

This is a crucial quote, which shows how, for Jason and Max, mixed-raceness was something that characterized California. Against this background, a mixed-race child would look “normal.” Ethnicity, race, state, and physical appearance were all merged in the expression “California look.” In the context of California, where a multiethnic look is perceived as desirable and the “future” of the state, which has a significant population from East and South Asia, Jason and Max were invested in what they called the “California look.” Their approach was not merely about familial resemblance but it reflected a desire to “blend in” to a look that has been marketed in this region. This way, they said, the child would reflect the mixed-race heritage that characterized the state in which they were living.

The multiethnic majority and the strong Latinx presence in California makes it possible for Latinx people to occupy a prominent place in the local hierarchies of beauty. This background may have been relevant to the fact that a single White Jewish dad through surrogacy interviewed in this study in San Francisco, Phillip, chose a Latina egg donor. Phillip, a 52-year-old freelance photographer, said he felt attracted to Latinx men as sexual and romantic partners, that is why he chose a Latina egg donor. On the contrary, for the two single White men interviewed in our study in England, the choice of a White egg donor was obvious.

The cases of the men in our research also showed how racialized categories were mobilized in malleable and surprising ways. For example, in Jason and Max’s thinking, some categories, such as “Caucasian,” were incorporated from the vocabulary used by fertility clinics. Other terms, such as “California look,” were created and racialized.

In contrast to the choice of egg donor, in the choice of surrogate, race seemed less relevant. The surrogate’s skin color and ethnic identification were not crucial for most men in this study. However, the politics of race also became apparent when Jason and Max were first matched to a gestational carrier of Native American descent. This would potentially raise issues since, as Max said, “any child born of a Native American in the US is a member of the tribe, and so in adoption of that child, the tribe has to be given the first right to adopt.” At least in this one case the history of colonialism, cultural genocide and the structural racism that disrupted the family formation of Native Americans, becomes apparent. The ongoing segregation, impoverishment, and legacies of genocide, and the forced assimilation and adoption of Native American children (Russell, 2018; Twine, 2017) are reflected in the most recent adoption law, in a move against white supremacy.

The primary logic of racial matching that Jason and Max followed was their search for racialized resemblance between themselves and their children. This logic was also used by almost all the families in this study, regardless of their own racial identification and geographical location. But Jason and Max also referred to another logic: resemblance between the child and the racial majority in their state. They found the multiracial context of California supportive for their multiracial family. This logic was mentioned only by some of the study participants, such as Kevin and Arthur in England, whose case we discuss at the end of this article. However, for most of the men who referred to this logic, it was secondary. Instead, the primary objective for almost all the interviewees was achieving racialized resemblance between themselves as the fathers and their child(ren)—and in some cases also among their children as siblings. Resemblance between the fathers and the children was also the aim of Luke and Jack, a multiethnic couple in England whose case we discuss next.

## Multiracial like us: an immigrant multiethnic/interracial couple in London

Luke, a 32-year-old brown-skinned manager at a media company, identifies as Australian-Filipino. Jack, his husband of 5 years, is a 30-year-old manager at a media company and identifies as a White Australian. Their daughter was 13 months old at the time of the interview. They met a decade earlier in Sydney and later moved to the United Kingdom as a couple. Luke is actively involved in the LGBTQ community at his company. As UK residents, they traveled to Canada to engage in an altruistic surrogacy contract to form a family. They decided to employ assisted reproduction, in part, because they believed it would allow them to prioritize the genetic links and increase physical likeness between themselves and their future child. Like other couples, both heterosexual and same-sex, they understood that skin color hierarchies and racial discrimination would have lifelong consequences for their children. Skin color is one feature that is “read” by others and assigned meaning and value (Hunter, 2005; Nakano Glenn, 2009; Thomas, 2009). Skin color, eye color, hair color, and other features are not a simple reflection of biological relatedness but socio-political legacies of colonialism.

Their pre-conception planning included how to maximize the probabilities that their child would share physical traits of both parents. A primary concern that Luke and Jack shared in the interview was how they would be perceived as a family unit. They wanted to avoid the emotional harm of not being publicly recognized as a “natural” family unit. This was also an issue among heterosexual multiracial couples in the United Kingdom. In her research in the United Kingdom among multiracial families not using assisted reproductive technologies (ART), Twine (2010) found that parents identified being questioned or challenged about their children’s racial origins as a source of stress and shame, and as a challenge to their respectability.

Luke and Jack decided that Jack would be the genetic father of their first child. This decision was motivated by the fact that none of his siblings had children, which had denied his parents of grandchildren. As Luke explained,

I have, like, five grandkids in my family already, huh? So my siblings each have kids, whereas my partner didn’t have kids, grandkids in his family. So that’s, that was really like what made the decision. Actually, . . . so when we got to the fertility clinic to do the sperm donation, we had every intention of fertilizing the eggs 50 - 50. Well, what transpired was we didn’t get as many eggs as we wanted. So there was only like seven. And I just thought, you know, like, to maximize the chance of, like, my partner’s shoot having a successful pregnancy, let’s just use his.

Since Jack identified as a White, the couple wanted a Filipina egg donor who shared Luke’s Filipino traits. This would help them create a mixed-race child. To their surprise, their daughter looks, in Luke’s terms—“purely Caucasian.” In response to the question about this decision who would be the egg donor, Luke said,

Just because we knew that we were likely using Jack’s sperm and he’s Caucasian, we really wanted the child to look like me a little bit, so I mean like a Filipino. And there were very few Filipinas in Canada, but we did find one. . . . So the funny thing is that it completely backfired because our daughter looks purely Caucasian, not Filipino at all! And we later found out our egg donor actually isn’t like a full Filipino, she has like a Caucasian grandma, and she’s got like some, like East Indian in her as well. So she was actually like, quite mixed, but she identifies as Filipino.

In this case, we can see that the racial logics embraced by Luke and Jack appear to be a product of binary thinking that assumes one’s physical phenotype reflects “discrete” and separate racial categories. As an interracial couple, Luke and Jack were committed to producing what they thought of as “likeness.” Using their logics, they treated socio-political categories such as “Caucasian” and “Filipino” as if they reflected “natural” biologically pure categories. So phenotype was assumed to follow genetic heritage. Luke expressed contradictory logics. Reflecting upon how he and his husband ended up with what they call a “Caucasian” daughter, he saw the hubris in his thinking. He now claims that being a parent is a “social” not biological relationship.

[Having a White daughter] is hilarious because that’s exactly what has happened! And it isn’t an issue. *I couldn’t care less what people think.* . . . being a parent isn’t about, like, a genetic connection. You don’t need to look like each other. *But this is something that I think I’ve, I’ve learned after the fact.* Yeah. Because we of course, start with some, you know, ideas and then as you, as you go, you see it in practice.

Ironically, this is still an issue. Luke and Jack are contemplating using the frozen embryo produced with the help of the Filipino egg donor they used in their first pregnancy and with Jack as the genetic father. If that embryo is viable, then their future child will be genetically related to its sibling. If that embryo is not viable, then according to Luke they might as well “flip it” and “try a Caucasian donor” with Luke as the genetic father:

We’ve got one [frozen embryo] as a backup. (. . .) we’re keeping it on ice, it costs like 400 bucks a year, so it’s not too expensive. We’re just going to keep it on ice in the event that we have, like, a sibling journey down the track. And I think the idea is, like, we’ll try and use that. And if it doesn’t work then we’ll go through the process of, like, finding an egg donor again, this time we will flip it, so we’ll try to get a Caucasian donor and use my sperm.

This showed that the couple’s thinking about genes and race was full of open questions. The same referred to the very meaning of genetic links between the parents and the children. On one hand, Luke said that having a family meant to him having a genetic connection. On the other hand, not only was he himself the non-genetic father to his child but he was also considering becoming a sperm donor for a friend, as he mentioned later in the interview. The meanings he gave to genetic links were thus multiple and sometimes apparently contradictory.

Like the majority of the men who participated in this study, Luke and Jack wanted their child to physically—and racially—resemble them. However, likeness between the child and the dominant White racial group was not important to them. This was also the case for most of the other multiethnic couples in this study (such as Jason and Max in California, Case 2 discussed above), as well as the White fathers (such as Bob and Mike in California, Case 1 above). Almost all of them prioritized achieving racialized resemblance between the fathers and their children, both as a proxy for kinship and a strategy against stigmatization. This included multiple dimensions such as the parents’ and children’s feeling of belonging to the family, and the display of such belonging to other people.

It was recapitulated clearly by Paul, a Black Caribbean-British 37-year-old marketing professional, married to Federico, a 42-year-old White Italian real estate professional. At the time of the interview, they lived in London, had a young son through surrogacy in the United States, and were in the process of another transnational surrogacy arrangement. They used the same mixed-race egg donor, who was half-African-American and half-Italian. Paul explained,

Although our children would be true siblings, we wanted them to look similar as well and look like they both belong to us . . . To keep this kind of resemblance and connection, for a sense of belonging, and also, you know, for cohesion with our family unit. You know, it’s very superficial, but it’s also something that can be important for children in terms of representation, and belonging . . . Or excluding one parent . . . I know, it’s very superficial. It’s quite an important consideration when you have a setup like this and a family structure like this. . . . That’s what I mean by kind of a cohesive family unit. So if you looked at us all, you would think, you know, that that’s a good set, you know, you can’t tell immediately what’s happened or who’s done what, and who’s donated, what sperm or whatever, because that’s where you start to invite really tricky questions, or people start to treat the children slightly differently.

Most multiethnic couples, such as Paul and Federico, or Luke and Jack with whom we started this case, seemed to care above all about creating kinship between themselves and their children. Yet they were not indifferent to the fact they belonged to a racial minority. Paul was part of a global Facebook group for Black gay dads, which, as he said, offered him a safe space to discuss unique issues his family could face, such as racism. Some other interviewees took a step further: they aimed to prevent racism at the early stage of the choice of the egg donor. This was the case of Kevin, the Latino gay dad in England, whose case we discuss below.

## Fast track to whiteness: a multiracial couple in England

Kevin, a 42-year-old brown-skinned man who identifies as Latino,^8^ grew up in a working-class immigrant family in New York. He moved to the United Kingdom in 2000. He met his husband, Arthur, a 40-year-old White British citizen, who grew up in a middle-class family in southern England. They are the parents of a 3-month-old daughter. They have been together for 20 years, and married since 2013. They both agreed that they wanted their child to know her origins. They ultimately used a surrogate who was also the genetic mother, which is now referred to as “straight surrogacy” (which is called traditional surrogacy in the United States) rather than “host surrogacy” (known as gestational surrogacy in North America). Kevin, the Latino dad, decided that he had a strong preference for a White child.

In contrast to Luke and Jack, the multiracial couple in the United Kingdom described earlier, Kevin’s logic was that he could protect his future child from racism or what he understood as racial abuse in the United Kingdom, by not having a child racially marked as “not White.” So they chose to use the eggs of a British woman who was classified as White. This decision was motivated by Kevin’s experiences with racism. He believed that his not being physically qualified for “whiteness” would be a non-issue if his husband and future child were both White. So his thinking was that a White child would blend in or be accepted more easily by his White husband’s family and broader society. In contrast to the California couple we met earlier, the “multiracial look” is neither celebrated nor normative in the United Kingdom even in multicultural urban communities, as it is in California. As we have seen in other couples, their pre-conception decisions were shaped by notions of “racial difference” and the social realities of racism. However, what constitutes a problematic difference varied.

In the case of Arthur and Kevin, it was not the White partner who preferred a White child. It was a familiarity with everyday racism that led Kevin to convince Arthur to be the genetic father. Moreover, Kevin thought, the child might feel more included in the day-to-day extended family if she shared some looks with them—and, as it later turned out, race/ethnicity was part of it. As Kevin explained,

We have a very strong connection and relationship with Arthur’s family [in the UK]. Arthur’s sister’s children are born through an anonymous donor . . . So, so this is a family where your children come into the world in very different ways. And there’s real love and compassion in making those connections with their family, based on, you know, the things that the older generations see in the children about, you know, oh, “you look . . . that looks like the grandfather,” or, you know, they love those connections. For me, having my dead parents and my family living so far away, it was inevitable that our child wasn’t going to have those stories and some of the stories about my past.

The elision of class and race status was also part of Kevin’s narrative, as if through a White British identification one could somehow acquire a middle-class status more easily than through a Latino background. That Latino background, Kevin feared, would expose his daughter to racism that he had been facing as a member of the Latino minority in the United Kingdom. Describing the racial context in which they live, Kevin noted “I do recognize that perhaps, to a certain extent, did play a bit of a part in, in my decision, because *we are a very White, very British world*.” Kevin went on to explain the way he perceived and experienced racialization. He understood White privilege as a form of cultural and symbolic capital:

I live in and work in very White dominant institutions. And as a senior clinician . . ., it’s something that I have to, you know, I have to fight for, in a way in which I don’t think my daughter will, and, and so I will be, I would be lying if I didn’t say that somewhere deep down, and it’s, and I feel ashamed saying it out loud. You know, today, I went for her check, and my daughter is rather tall and rather long, for a baby. And I was just thinking, yeah, *I’m gonna have a tall daughter, who’s going to . . ., who’s going to be raised in an educated, middle-class family, who . . . she will have lots of opportunities*, and which is very different to my working-class, Latino roots, where, educationally I’m the only one that really . . . you know, I, I’ve done really well. But actually, my daughter has lots of privileges in a way in which I didn’t have. And and I do feel, I think the thing I feel ashamed of, and I do feel ashamed is that, that that did play a key part in some of this decision, even if I don’t talk about it, even if I don’t talk about it. I’m sure it did play a big part.

Arthur initially assumed that because they had selected a White English surrogate-egg donor, that his Latino husband would become the genetic father. Thus, he perceived their future “mixed race” child would reflect the racial-ethnic identifications of both fathers. Arthur also thought that they would each take turns as genetic fathers. Arthur eventually agreed with Kevin’s racial reasoning to privilege his White extended family. Kevin anticipates his daughter’s future struggles as the child of a Latino. He stated, “I feel sad that I had to say that or do that, in lots of ways.” Below we see the deep thought that Kevin has given to the intersecting forms of stigma that he is reflecting upon *before conception*:

I said to Arthur . . . the role of a parent is to make sacrifices and to do what’s best for their child. And, and I do see that my decision to not be the bio dad was a sacrifice, it was a sacrifice, because I also had to weigh up what world we live in, and I would like my daughter to have the best start in life. . . . to a certain extent, *that’s also recognizing oppression and marginalization, and she would be coming into a world where she still has two dads, if she’s coming to an interracial intercultural family*, she’s got my surname. So she has got a Latina surname, you know, she is going to have lots of different areas or aspects of her life where she’s going to have to fight, she is a woman in a world of misogyny. . . . She will still have a Latino father. (. . .) I know oppression and marginalization. *Fighting is tough. Fighting is exhausting, and I don’t want my daughter to have to fight any more than she has to*.

Here, Kevin clarifies that as a brown-skinned Latino he has personally experienced racism. He has decided that he does not want to transfer his status to any future children. Kevin and Arthur’s story suggests that the reproduction of whiteness and prior experiences with racism were the motivation for their decisions. Kevin made the assumption that as a resident of England, it would be easier for his daughter if he transferred White skin privilege to her, so that she could pass as English. Even if Arthur had initial objections, the couple agreed that likeness increased the child’s chances not only to become part of the extended family but also to have racial privilege in the United Kingdom. On the other hand, in contrast to England, in much of California Latinos are not a minority group. A half of the Latinx population in the United States self-identifies as White and their children are often perceived as White. Latino is therefore not a racial category, but an ethnic one that includes people who are physically eligible for whiteness.

## Conclusion: resemblance as a proxy for kinship and belonging

In this article, we have drawn on a transatlantic study of gay male parents to document the ways in which they undertake racial matching in the choice of egg donors in surrogacy arrangements in California and in England. Building on the work of Thompson (2005), Twine (2010), and Franklin and Roberts (2006), we called this process *strategic racialization*. The interviewed men undertook strategic racialization with the objective of managing racial transgressions, alongside the transgressions they already made as gay parents and users of surrogacy. We found that gay parents may encounter two types of stigma: (1) racialization as an interracial or multiethnic family, (2) being perceived as transgressive sexual dissidents, in national contexts where same-sex marriage has been legalized not long ago, in 2013 in the United Kingdom, and 2015 in the United States. Nevertheless, the legalization of same-sex family formation does not prevent gay men from experiencing marginalization. Consequently, in their pre-conception negotiations, they try to restrict potential forms of stigma by presenting themselves as a “natural” family. The gay male parents in this study sometimes embraced hegemonic ideologies by default. These are ideologies that translate cultural and physical differences into “natural” and biological categories based upon ideas that posit racial purity and, by extension, can support what some ethnic minorities experience as white supremacist aesthetic hierarchies.

In the process of strategic racialization, the men followed two racial logics. The primary logic used by almost all the families in the study, whether they identified as White or multiethnic, was the logic according to which the men sought to achieve racialized resemblance between themselves and their child(ren). This logic was used by almost all the men interviewed both in California and England. This points to the enduring relevance of traditional Euro-American kinship (Franklin, 1997; Thompson, 2005), as the men thought that kinship was enacted, reinforced, and inherited by racialized biogenetic links between parents and children. These gay parents’ perceived vulnerability can lead to their dependence upon racialized forms of “likeness” between themselves and their children. Some of the men also aimed to reinforce kinship by seeking resemblance between their first and their second child. One of the strategies to achieve it was using the same egg donor.

The other logic the men employed in racial matching was seeking resemblance between their child(ren) and the racial majority in their broader communities, such as their extended families or the states they were living in. That logic was usually secondary and only few of the men referred to it. Yet importantly, they were mostly multiethnic couples. They talked about the racial majority as a potential source of empowerment and support for their families and children, who could easily “blend in.” These men’s perceptions of risk and potential harm to their future children can, paradoxically, reinforce the locally dominant racial hierarchies. In situations like California, a “White” look is not always perceived as superior or desirable in the same ways as it might in the UK context. The men interviewed in California did not refer to the White majority as a model with which they would like to fit in. On the contrary, some of them referred to the multiracial “California look” and Latino look as desirable characteristics. On the other hand, in England, some of the interviewees mentioned that it would be beneficial for the children if they did not stand out from the racially dominant White society, institutions, and families. These findings suggest that construction and performance of race is context-dependent. It has unique characteristics in the families of gay men interviewed in California as compared to those interviewed in England.

Our findings represent the first study to offer a comparative analysis that illuminates and problematizes the strategies employed by gay men in the United Kingdom and the United States. Our findings also echo previous studies on racial matching by lesbian mothers and heterosexual parents (Davda, 2018; Mamo, 2007; Nordqvist, 2012; Ryan and Moras, 2017) as well as gay fathers (Murphy, 2015; Norton, 2018). Our comparative focus provides insights into the place of contemporary queer kinship within the transatlantic and post-colonial dynamics of Euro-American kinship. Our analysis of interviews with multiethnic couples also points to the increased scrutiny and pressures faced by multiethnic families in the context of the ongoing reproduction of whiteness.

## Notes

1. This research was conducted by Marcin Smietana.

2. With a population of roughly 40 million, California is the largest state by population in the United States. San Francisco, a city and county located in northern California is also one of the birthplaces of the Gay Pride movement of the late 20th century.

3. Leicester, a multiethnic city in the East Midlands of England, has the largest population of South Asian ancestry in Europe.

4. Some forms of altruistic surrogacy are also permitted in the Netherlands (including gay parents) and Greece and Portugal (only for heterosexual couples).

5. According to the 2011 UK census data, 86 percent of England and Wales (where the UK study was carried out) were White: https://www.ethnicity-facts-figures.service.gov.uk/uk-population-by-ethnicity/national-and-regional-populations/population-of-england-and-wales/latest

6.
https://www.census.gov/quickfacts/fact/table/CA/PST045219

7. All interviewees’ names are pseudonyms.

8. We are using the term “Latino” to honor and respect the wishes of this interviewee, who expressed anxiety and fears around racism. Kevin has clearly suffered as a hyper-minority and thus we are not using precise references to his country of origin. Following his guidelines, his story was de-identified as much as possible.
